# The Beat

**DOI:** 10.1289/ehp.120-a150b

**Published:** 2012-04-01

**Authors:** Erin E. Dooley

## April 1–7 Is National Asbestos Awareness Week

In March 2012 the U.S. Senate for the second year in a row approved Resolution 389, declaring the first week of April to be National Asbestos Awareness Week.^1^ Sponsored by Max Baucus (D–MT), the goal of the measure is to increase public awareness of the prevalence of asbestos-related diseases and the dangers of asbestos exposure. The National Toxicology Program declared asbestos a known human carcinogen decades ago.^2^ The United States, which no longer mines asbestos but still imports it,^3^ is one of the few industrialized nations where asbestos is not banned.

**Figure:**
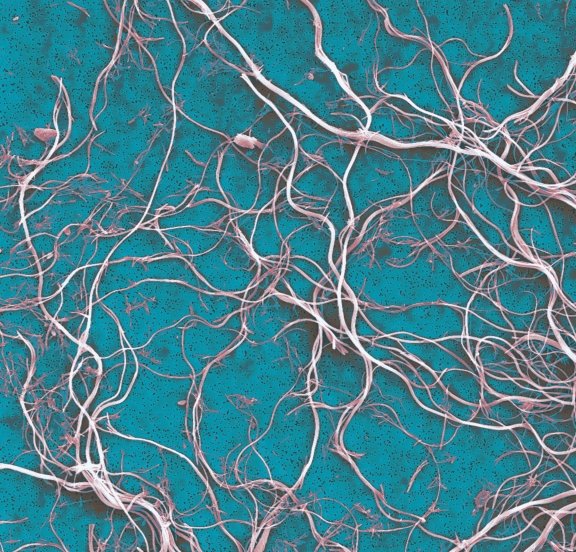
Asbestos exposure is the only known risk factor for mesothelioma. © Ken Lucas/Visuals Unlimited/Corbis

## Safe Drinking Water Target Met

With goal 7 of the Millennium Development Goals Declaration, the WHO and UNICEF pledged to halve the proportion of the world’s population lacking access to safe drinking water and basic sanitation by 2015. In March 2012 the WHO announced that part of this goal had been met—and exceeded—with the provision of sustainable access to safe drinking water to 89% of the population.^4^ Since 1990 more than 2 billion people have gained access to improved sources of drinking water, nearly half of them in China and India. More than three-quarters of a billion people still lack access to safe water.

## Tobacco Smoke Exposure in Utero Linked to Eczema in Infants

Exposure to tobacco smoke *in utero* has been linked with the development of asthma and respiratory infections in infants. A new preliminary study of more than 1,400 infants, presented at the 2012 Annual Meeting of the American Academy of Allergy, Asthma & Immunology, finds it may also affect the development of the skin condition eczema.^5^ Infants exposed during the third trimester were significantly more likely to develop eczema after birth, compared with unexposed babies. There were no increases for infants exposed during the first trimester or in the first 6 months following birth. Nationwide, an estimated 10% of children under age 18 have been diagnosed with eczema.^6^

**Figure:**
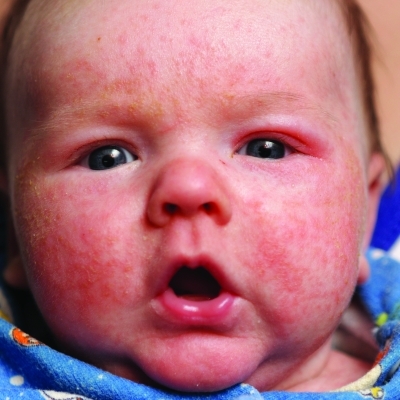
Prenatal exposure to tobacco smoke may contribute to eczema. Shutterstock.com

## Federal Government Announces New E-Waste Policy

The U.S. General Services Administration has announced new guidelines^7^ forbidding federal agencies from disposing of electronics equipment in landfills or incinerators. The guidelines ask federal agencies to continue to reuse electronics to the fullest extent possible before disposing of them at certified e-waste recycling facilities. Buyers of used government electronics will be encouraged to dispose of such products similarly. The guidelines align with sustainability goals set for the federal government by President Barack Obama.

## Timing of Spring and Fall Affected by Urban Heat Islands

The urban heat island effect has been shown to influence the onset of spring and autumn in some areas, but not others. In a new study using 25 years of high-resolution satellite data, researchers observed later autumns and, to a lesser degree, earlier springs in forests within 35 km of Washington, DC, and Baltimore, Maryland, compared with forests farther away.^8^ They report this is twice the distance observed in earlier studies using coarser-resolution data. Each year the slope of spring’s transition time typically correlates with that of the fall that follows. The researchers hypothesize that changes in plant communities and soil moisture attributable to urban development may explain why urban heat islands impact autumn’s timing more than spring’s.

**Figure:**
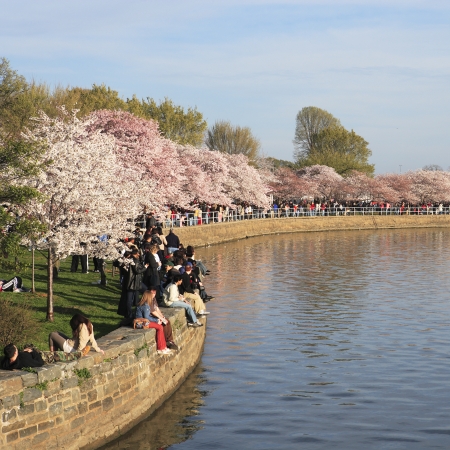
The urban heat island effect can hasten the onset of spring. Shutterstock.com
